# Book Reviews

**Published:** 1822-06

**Authors:** 


### Monthly Medical and Scientific Bibliography.

Vexat censura . . . . . . Corvos.

### 1. GREAT BRITAIN.

Art. I.—*The Nursery Guide; or, "Remarks on the Conduct of a Nursery:" containing Information likely to be useful to those who may shortly take upon themselves the Duties of a Mother; and particularly offered to the Notice of Females in general.* By Henry Thompson, Surgeon, Enfield, Middlesex. 8vo. pp. 64. T. and G. Underwood, London. 1822.

FROM the title-page of this little work, none of our readers would, we dare say, be inclined to expect a poetical treat. Yet so it is. The Naiads of *Enfield Wash* have inspired Mr. Thompson; nor are we less disposed to think well of his production because it is drawn up in measured lines.

There has been no haste evinced in publishing the little poem before us. Mr. Thomson was led, from the purest motives, to delay its publication; and we doubt not but, in the long interval that has elapsed since it was first written, the author has profited of many suggestions for its improvement.

The instructions detailed in this Guide relate to the conduct of a nursery; to the choice of a wet nurse,—to exercise,—moles,—flatulency,—thrush,—red gum,—teething,—convulsions,—worms,—small-pox,—chicken-pox,—vaccination,—eruptions,—scarlet fever,—watery head,—scrofula,—Dalby's carminative,—gin,—advice,—bathing, and early hours.

These multifarious objects are treated in verse by Mr. Thompson; a language he thinks likely to attract more the attention of the fair sex, and one which seems more appropriate to convey salutary lessons to the tender mother, full of solicitude for her new-born babe.

Of course, the goodness of the advice is to be sought, in such a production as this, more than the terseness of the line, the bright thought, the lucid order, the fire of poetical genius, the pleasing metaphor, the elegant and soft-gliding diction. A poem for a nursery, which is to teach when to put a baby into the hot bath, or when to give it what Mr. Thompson has very happily termed "Turkey's far-famed plant aperient," may do without all these essentials of a poem, provided, as we have already asserted, the advice it conveys be good. That it is so, and properly given, we can take upon ourselves to say; but, whether the music-strains in which that advice is conveyed be equally good, we must leave our readers to judge, either from the poem itself or from the following specimens:

"FLATULENCY.

"The frequent pains which infants feel from wind,
Are by those evils, heretofore remark'd,
Of faulty diet caused; and not, as oft
The misled nurse describes, from acid, bile,
Or such like nonsense, in her brain alone.
Children are seldom born with a disease:
But, as we envied thus their happy lot,
We early seek to make them share, forsooth,
By every art, those we ourselves possess:
And not unfrequent do the writhing forms
Of anguish'd limbs, in attitudes of pain,
Spring from our senseless selves; whilst eructations,
And countenance descriptive of distress,
In pity make us feel those ills our own,
When we *can feel* for any thing but self."

"WORMS.

"Another fevering form of ill exists,
Exciting symptoms similar to those
Just mention'd: worms, infesting the canal
Intestinal; these oftentimes produce,
Though from a diff'rent cause, the same effects,
(As far as *cursory observation* shows,)
Yet are they vastly diff'ring, whilst no change
Is seen in some, so do they vary oft.
The symptoms which proclaim their sick'ning pow'r,
Are parching fever, itching of the nose,
The swollen belly, 'yond th' accustom'd size
Of health's establishment, with peevishness,
And sleepless weariness, and appetite
Of most vorac'ous insatiety;
Drought and much pain untold create the flame;
The hectic flush succeeds the pallid white,
And lily paleness hunts the roseate bloom.
Fretful, impatient, tired of itself,
Is the poor suff'rer, heedless of constraint,
In its misdoings still persists, nor heeds
Reproof, though properly applied;
Peevishly loth, unwilling to obey,
With temper irritable, fractious grown,
Each word is contradiction, anger, rage;
Until, with passion breathless, quite worn out,
It sinks th' exhausted victim of its ire."

The work is interspersed throughout with several judicious prose notes.

**Art. II.**—*The London Dispensatory: containing, 1, the Elements of Pharmacy; 2, the Botanical Description, Natural History, Chemical Analysis, and Medicinal Properties of the Substances of the Materia Medica; 3, the Pharmaceutical Preparations and Compositions of the Pharmacopœiæ of the London, the Edinburgh, and the Dublin Colleges of Physicians. The whole forming a Synopsis of Materia Medica, Pharmacy, and Therapeutics.* Illustrated with many useful Plates, Tables, and Copper-plates. By A. Todd Thomson, F.L.S. &c. &c. Third Edition. pp. xvi. 962. London, 1822.

The character of this most excellent work, like that of its accomplished author, is so well known, and so firmly established, that we merely notice it, to afford an opportunity, to those who are anxious to see the progress which pharmacology is every year making, of procuring the third edition, wherein much that is new will be found, both in the text and the notes, ably developed. Mr. Thomson's very extensive reading has enabled him to collect a body of information, in the present edition, equalled in none other of the contemporary publications of a similar nature.

To render this Dispensatory still more useful to the generality of practitioners, the author has added, in the Appendix, four well-written, concise, and historical papers, on as many subjects of great practical importance, now engaging much of the public attention. We allude to the hydrocyanic acid,—iodine,—oil of croton,—and the wine of colchicum.

The introduction of the first and the last of these medicinal preparations is now so firmly established, that a notice of each from the author of the London Dispensatory became indispensable. The other two articles are yet subject matter of speculation, on which the opinion of medical men is much divided. We were glad to find a notice of them in this work.

Among the very many additions and illustrations contained in the present edition, we may also mention that Mr. Thomson has inserted, from the popular work of Dr. Paris, all the information regarding the composition of those quack, or, as they are termed, patent medicines, which he has considered useful; "for," observes Mr. Thomson, "as the author of the Pharmacologia has done me the honour to extract very largely from the Dispensatory, I have, in return, not hesitated to borrow a little of the curious and novel matter which his notes contain." This is all fair. Books are public, and consequently common property. That authors should borrow the ideas, doctrines, conclusions, and facts, of their predecessors or contemporaries, is a circumstance at which we ought to rejoice at all times; but, in fairness to the original, as well as in justice to their own characters, copyists should acknowledge the source from whence they have borrowed their information. Since we introduced one of the articles, mentioned by Mr. Thomson in his Appendix, into the medical practice of this country, and more especially since the publication of our historical treatise on that medicine, the information contained in that book, and by us communicated for the first time to the public, has been pirated in every direction, without the least acknowledgment, or the mention of our name. How different the practice of an honest and candid author like the one before us; an author who can boldly stand on his own great merits, and disdain borrowed plumes!

---

**Art. III.**—*An Answer to the Review of the 6th Edition of* Dr. Thomson's *System of Chemistry in No. XXI. of the Journal of Science, Literature, and the Arts, edited by* Mr. Brande. By the Author of that System.

Some people are decidedly incorrigible! Mr. Brande, we should have thought, had received, at our hands, such a recent castigation, when the itch of criticizing his betters again seized him in April 1821, that we should have expected he would have listened to the dictates of prudence or the suggestions of friends, and held his tongue as a chemical reviewer. His evil star, however, prevailed; and he had no sooner emerged from the mire into which a certain "Reply" had thrown him, than he contrives to get into it again,—"deeper and deeper still!"

Mr. Brande, from the first moment of his illustrious predecessor yielding the chemical chair at the Royal Institution to him, seems, positively, to have forgotten his relative position in society. Instead of pursuing the path of industrious research for the promotion of science,—of which disposition we really thought he had, at first, given favourable indications; and for doing which he is paid by the members and supporters of the Royal Institution,—he betook himself to the office of censor of public institutions and of private individuals. But with what stock of information, with what qualifications, does the public think that he came forward in that capacity? Without, as Dr. Thomson informs us, the benefit of a university education,—that is, without that which is considered, both in this highly civilized country, as well as in every other civilized country in Europe, not excepting even that of which Mr. Brande's father was a native,—the only great distinction which can characterize people who are anxious to be reckoned among the scientific and the literary.

What the results of such an undertaking have been to Mr. Brande, the public may easily learn by reading over the periodical publications, both foreign and domestic, for the last eight years. Mr. Brande's name is there quoted,—not as the disco-

5

verer of new facts, the inventor of useful apparatus, or the expounder of obscure doctrines, as we had hoped to see, and had a right to expect from him; but as the combatted and the discomfited censor of long-established and valuable institutions, or of authors equally eminent for their private worth and their skill in science. The professor first directed his attack against the Royal College of Physicians in London; and, in Dr. THOMAS YOUNG, (*Ciel! quel contraste de noms!*) one of that learned body, he found a tremendous antagonist. He next pointed his shafts of criticism against the universities of Oxford, Cambridge, and Glasgow, with the systems of which he proved himself wholly ignorant; and we need not mention how triumphantly he was defeated, on those occasions, by the able replies of the professors of those institutions. Not satisfied with this, he drags forth to unmerited derision two of the first characters of the age, whose names are become as classical as their labours, BERZELIUS and GAY LUSSAC; but the attempt was so puny, that the laughers were all, on that occasion, against him. Two such men needed no defence against the editor of the Quarterly Journal of Science! He then fell headlong upon the author of the best system of chemistry extant; and, by inserting in his Journal a most virulent attack against him, both as a man and an author, calls forth the animadversions of the good, and the tardy, but damning, reply of the author himself, which we now announce.

Our readers know that the editor of the Quarterly Journal had, before this last *exploit*, made a most unjust, unprovoked, and shameful attack against us, poor humble individuals, compared to the men we have just mentioned,—against us, then in habits of intimacy with him,—against us, his coadjutors in compiling the Journal of Science,—against us, who were in the habit of supplying him, exclusively, with all foreign information, and the analysis of foreign works and Journals. Whether that attack was successfully repelled,—its misrepresentations exposed,—its fallacies detected,—the ignorance it betrayed pointed out,—and its real motives ascertained, we leave to the numerous readers who perused the "Reply" to decide.

Just such a glorious termination has the present contest had, between Dr. Thomson and Mr. Brande. The answer we now announce (and which, besides being printed in the Annals of Philosophy for April, has been widely circulated in a separate form,) does so expose the wickedness of the attack contained in the 21st Number of the Journal of the Institution against Dr. Thomson, that we should hope never to see the pages of that Journal sullied again with such performances. Let the members of the Royal Institution look to this. Are they to continue to lend their name to a Journal which has so often become the vehicle of abuse and slander against persons whose aim and care is, conscientiously, to promote the advancement of science!

One of the observations of Dr. Thomson's reviewer savours so much of ignorance, that we cannot refrain exposing it. He asserts that "the English have, at least, six respectable chemical compilations by different persons, while the French nation are satisfied with one treatise—that of M. Thenard." Are not, then, the other French elementary works on chemistry equally respectable with the six English works alluded to by the reviewer; or was he ignorant of their existence? Why are Lavoisier's System, Fourcroy's two elementary works, Chaptal's, Bouillon Lagrange's, Fabulet's, Orfila's, Pelletan's, and many others—new editions of which are very frequently printed to supply the increasing demand for elementary books on chemistry—passed over in silence? We have no hesitation in saying, that, in chemical literature, in elementary and practical works on chemical science, the French, far from being behind us, are decidedly our superiors.

It will be sufficient for us, in this place, to remark, that almost every charge brought against Dr. Thomson is successfully rebutted by that gentleman, who thus concludes his reply:

"Thus have I finished my remarks on Mr. Brande's review of my System of Chemistry. When I perused it, for the first time, with attention, in the month of February last, the impression which it left upon my mind was, that many of the animadversions must be well founded. They are made with an air of such confidence and plausibility, that they are well calculated to make an impression on the reader. After having thus investigated them one by one, I am amazed to find how very few of them have any justice in them; and feel fully confident that every reader will participate in my astonishment, and agree with me that a more uncandid review has scarcely ever appeared, and that it fixes an indelible stigma both on the editor and the author."

---

### 2. FRANCE.

Art. IV.—*Traité des diverses Amputations qui se pratiquent sur le Corps Humain, &c.* Par le Dr. Maingault. 1 vol. folio. Paris, 1822.

This is an excellent idea, and another of the useful applications of the lithographic art, for which we are indebted to our ingenious neighbours.

Each different amputation is faithfully represented by drawings, on stone, taken from nature; the different steps of the operation, and the manner of performing it, being pourtrayed in the most accurate and satisfactory manner. We commend, above all, the great exactness of the designs, and the precision of the chirurgical descriptions that accompany them. Baron

Percy, who must be allowed to be an excellent judge in these matters, has made a highly favourable report of the work to the Royal Institute.

To those young surgeons and country practitioners, whose occupations afford few opportunities of seeing the principal operations of surgery performed, this work will prove of the utmost utility; and, as a guide, the best they can possess.

---

Art. V.—*Histoire des Phlegmasies, ou Inflammations Chroniques, fondées sur de nouvelles Observations de Clinique et d'Anatomie Pathologique.* Par F. I. V. Broussais. 3 vols. 8vo. Paris, 1822.

If the rapidity with which the editions of M. Broussais' book succeed each other should go on increasing at the present rate, and the author keep adding a fresh volume to each new edition, he will soon have outstripped, in number of volumes, the far-famed *Dictionnaire des Sciences Medicales.*

The present is the third edition of a work, of which the first appeared in 1808 and the second in 1816. That it contains a vast collection of facts, from which many useful inferences may be drawn, cannot be denied: but we have always, and must, to this moment, deny that it embraces the only real and correct views in medicine which practitioners ought to follow.

The edition we announce contains several important additions.

---

Art. VI.—*Faune des Medecins; ou Histoire des Animaux et de leurs Produits considerés sous le Rapport de la Bromatologie et de l'Hygiéne en general, de la Therapeutique, de la Pharmacologie, et de la Toxicologie.* Ouvrage entièrement neuf, par H. Cloquet.

This is the title of a projected work by a gentleman who has distinguished himself in the pursuit of natural science. It is intended as a sort of medical Zoology, in which not only the natural history of those animals that are interesting to the health of man is fully given; but also every particular connected with them is detailed, which is referrible either to the composition of medicines, or to the preparation of food for the human species.

We prefer letting the author speak for himself as to the nature of his undertaking.

"Son livre est destiné à offrir l'histoire spéciale de chacun des animaux qui peuvent intéresser, en quelque point, la santé des hommes, et à présenter des détails sur leur *synonymie* et même sur l'*étymologie* de leur nom, sur l'ensemble de leurs *caractères* et des propriétés qui les distinguent et doivent servir à les faire reconnaître, enfin sur les lieux qu'ils habitent, détails qui appartiennent à tous sans exceptions.

"Mais il est de ces animaux dont la thérapeutique implore le secours: alors, à ces premières données viennent se joindre des pré-

ceptes sur la manière de s'en emparer et de conserver celles de leurs parties qui sont utiles à l'art de guérir; sur les préparations qu'on doit leur faire subir; sur les doses auxquelles on les administre ; sur l'action physiologique qu'elles exercent dans notre organisme; sur leurs propriétés médicinales, réelles ou supposées ; sur les falsifications qu'on peut leur faire éprouver, et sur la manière de reconnaître enfin celles-ci.

" D'un autre côté, le médecin n'agit point seulement à l'aide des médicamens; le régime devient une arme puissante entre ses mains. Il ne trouve donc pas moins d'intérêt dans l'étude de tous les animaux qui contribuent à l'alimentation de ses semblables, et en particulier dans l'examen attentif de cette vingtaine d'espèces si importantes dans l'économie générale de la Nature, et que nous avons su réduire à l'état de domesticité pour profiter de la chair, du sang, du lait, des œufs, du beurre, &c. qu'elles fournissent journellement à nos besoins. On conçoit sans peine à combien de considérations utiles une semblable matière peut donner naissance.

" Ce n'est point tout encore. Il ne suffit point de connaître la sangsue, les cantharides, la civette, le musc, les méloës, les mylabres, consacrés au soulagement de nos maux ; ou le mouton, la poule, la vache, le porc, destinés à assouvir notre faim et à réparer les pertes de notre organisation : il existe des animaux possesseurs d'un poison actif, qui répandent autour d'eux la douleur et la stupeur, et qui quelquefois même, après avoir cessé de vivre, peuvent faire encore germer dans notre sein le funeste principe d'une mort assurée. Le médecin doit les connaître ; il est souvent appelé pour combattre l'espèce d'empoisonnement auquel ils donnent lieu ; rien de ce qui les concerne ne saurait lui être étranger ; le tableau des symptômes qu'ils déterminent n'est pas moins important pour lui, que la science des remèdes propres à faire cesser les accidens effrayans qu'il a à vaincre lui est indispensable."

The work will be completed in thirty numbers, each composed of six sheets of letter-press and two plates, coloured, price three francs. The first number will appear on the 1st of July.

---

### 3. PRUSSIA.

ART. VII.—*Beitrage Zurnähem Keutroff, &c.—or, an Attempt to improve the Knowledge of Sporadic Typhus, and some other Diseases connected with it, by Necroscopical Inquiries.* By C. F. POMMEN. 8vo. pp. 140. Tubrughen, 1821.

FROM the author's researches, it would appear that sporadic typhus is neither constituted by an inflammatory state of the brain, nor of the nervous system. It has seldom happened to him to witness any sign of inflammation, after death, in either of those parts of the animal economy. The pectoral as well as the abdominal viscera, on the contrary, have invariably exhibited the morbid alterations caused by inflammation ; so that the morbid process by which sporadic typhus is constituted appears to extend to many organs and systems at the same time.

The author adduces the doctrine of *inflammationes occultæ*, invented by the ancients, as one which approximates, nearer than any other, to any thing like a satisfactory explanation of the proximate causes of typhus. A state of irritation, simulating inflammation, may subsist in this disease; or a degree of erethismus of the brain may prevail through the sympathetic influence of distant organs; in both which cases, blood-letting may prove injurious.

The author, therefore, recommends the cautious use of this therapeutical means, and prefers the employment of bland purgatives, mucilaginous drinks, local depletion, and mercurial frictions over the surface of the abdomen; due regard being had to the strength of the patient, and the approach of a crisis in the disease.

---

### 4. PORTUGAL.

Art. VIII. —*Memoria sobre os Meios de Diminuir a Elephantiase em Portugal, &c. offerecida a's Cortes de Portugal.—Memoirs on the Means of checking the Prevalence of the Elephantiasis in Portugal.* By Dr. A. B. de Gomes. 4to. Lisbon, 1821.

The *lepra*, a disease of such rare occurrence amongst us, prevails to a great degree in Portugal. The author, who is a member of the Board of Public Health, has discharged the duty of a good citizen, in calling the attention of the legislature to this point. Amatus Lusitanus, Rodrigues a Fonseca, Zacutus, and other Portuguese physicians, had already seen this loathsome disorder in Portugal; but it was then much less frequent than now. The provinces of Minha, Beira, and others, are infected with it. In the capitanshire of Minos Geraes, at the Brazil, it also prevails. In the hospital of St. Lazarus, in Lisbon, there are, at this moment, from twenty-five to thirty cases of elephantiasis in men, and from eight to ten in women. M. Gomez reckons that there are, at least, eight hundred *leprosi* in different parts of the kingdom.

As to the means proposed by the author for extirpating this disease, by the establishment of specific hospitals or lazarets, *(gafarias,)* they are by far of too local a nature for us to notice them in this place.

A theiform infusion of the *arum colocasia*, previously roasted and powdered, has been strongly recommended in this complaint; but the result has not been so satisfactory as that obtained from the use of arsenic, agreeably to the practice followed in India. The use of colchicum and prussic acid have also been proposed, and had recourse to; but hitherto, as it would appear, with little success.

### 5. HOLLAND.

ART. IX.—*Considerations pratiques sur la Maladie des Femmes en Couche connue sous le nom de Peritonite et de Fievre Puerperale.* Par J. B. VANDERZADE, Physician to the General Civil Hospital, at Antwerp. 1 vol. 8vo. 1821. Antwerp. pp. 129.

THERE is a singular circumstance connected with this work which leads us principally to notice it. The author admits that the nature of the disease on which he has undertaken to write is perfectly well known and correctly understood. He himself admits that it is purely and distinctly inflammatory; but he, at the same time, insists that neither blood-letting nor the application of leeches, now so prodigally resorted to in the treatment of that complaint, are proper, salutary, and justifiable means. But for the application of his doctrine to the complaint under consideration, in which M. Vanderzade is decidedly wrong, we would willingly admit the correctness of his general remark against the present mania of seeing nothing but inflammation in every case of disease.

"En reconnaissant," says M. Vanderzade, "que la maladie dont nous nous occupons, est une inflammation de peritoine, nous ecartons du traitement et la saignée et les sangsues que l'on voit prodiguer maintenant, avec l'eau gommé et l'eau sacrée à la moindre douleur du ventre, de la poitrine, ou de la tête, comme si le genie inflammatoire presidoit à toutes les maladies, et qu'il n'y eut pour le combattre dans tous les cas, qu'a degorger *les vaisseaus sauguins toujours coupables de plenitude et chargés de l'anathême qui les livre à des myriades de sangsues dont la population*, si cela dure, est menacée de s'eteindre."

The author having discarded bleeding and leeches from the treatment of puerperal peritonitis, found himself obliged to look out for some more effective remedial agent with which to combat that disorder. This he has found in *calomel*, from the use of which he pretends to have obtained more benefit than from any other measure adopted in such cases by the generality of practitioners. We scarcely need dilate on this subject with English readers, who must long have been familiar with the practice now brought forward as a discovery; but we cannot suffer him to quit us without giving him a piece of useful information—that if he goes on relying on calomel alone in puerperal inflammation of the peritoneum, his future success will greatly disappoint his expectations.

### 6. ITALY.

Art. X. — *Nuovo Saggio Analitico sull' Inflammazione.* Del Cavaliere Giuseppe de Filippi, m.d.—*A new Analytical Essay on Inflammation.* By the Chevalier Joseph Filippi. Milano. 1821. 8vo. pp. 236.

Of the real nature of inflammation we have as yet no precise notion. In the treatment of it we have lately made very great improvement; the continental practitioners still more than any other, particularly those of Italy and next those of France; for after bleeding they do not prescribe the compound aloetic, or gamboge pills, or the cathartic extract, with a prodigious black dose, containing tinctures, aromatics, compound infusions, and Heaven knows what other fiery drug. This question, as far as it regards inflammatory complaints, being brought to an issue, the public may rejoice at the result; for if we are now successful in combating inflammation, it imports not a straw, whether we know, or know not, what is the morbid process in nature, by which inflammation is produced.

We see, therefore, with regret, an attempt made by the present author (in every other respect a most worthy practitioner) to throw us back half a century in regard to the *methodus medendi* in sthenic maladies. He is horror-struck at the profusion of blood, at the enormous quantities of tartar emetic, antimonials, calomel, digitalis, prussic acid, squill, ipecacuanha, with a few more articles of the contra-stimulant system so liberally prescribed. To check such a practice, he comes forward with all his experience and erudition; and endeavours, by reasoning as well as by the exposition of facts, to point out the mischief likely to arise from the prevalence of the present doctrine of universal inflammation.

Dr. Filippi first describes what he considers inflammation to be; and next watches the progress of this morbid action through its various effects on the animal system.

The first of these he calls "Lesione idraulica del circolo;" next to it comes suppuration—serous and purulent effusion; enlargement, induration, atrophy, softening, disorganization, gangrene.

This analytical history of inflammation is very ably drawn up, and seems founded on principles of the soundest pathology.

Vitality is a principle which both affects and is affected by inflammation. It was natural, therefore, for Dr. Filippi to enter into the consideration of this entity. This question is fully debated in the third chapter; and we were pleased with the manner in which the author has conveyed to his readers his own conception of the organization of the human body.

"L'organizzazione del corpo umano io la ripeto da tre elementari tessuti: il tessuto nerveo, il tessuto vascolare ed il tessuto membranoso. Il primo fabbrica e dispensa la vitalità: il secondo provvede i materiali per essa vitalità e pella organizzazione; il terzo lega, raccoglie e modella i due primi tessuti e costituisce la compage organica dell' essere intiero. I sistemi osseo e muscolare non sono propriamente organici ed elementari, poichè derivano dai tre primi e sembrano unicamente destinati ad estendere la sfera della vita animale. Osservasi che nella fabbrica delle parti e degli organi, que' tre primi elementari tessuti si intrecciano e si confondono sì fattamente tra loro, che ove si trovino vasi, v' abbia e nervi e tessuto membranoso o cellulare, così viceversa. Quindi è più facile l'immaginare l'esistenza di questi tessuti organnizzatori che possibile riscontrarli nel loro stato semplice ed elementare. Ma per complicati e confusi che sieno fra di loro questi tessuti, ogni parte ed ogni organo ritiene il marchio del tessuto predominante e fondatore, per modo che i suoi attributi dipendono essenzialmente dalle proprietà vitali inerenti ad esso tessuto. Da qui ne viene la varietà delle forme vitali che si osserva nella economia animale; e si può dire, con un celebre fisiologo francese, che le parti e gli organi, oltre alla vita generale, possegon tutti una particolare attitudine di esprimere i loro attributi.

There are some excellent remarks in a subsequent part of the work on the necessity of taking into consideration the structure as well as the importance of the different tissues or organs affected by inflammation; and these are followed by the author's classifications of inflammatory modifications. The annexed extract will convey a clear idea of his particular views on this subject.

Le forme del processo flogistico, siccome abbiamo ripetutamente annunciato, desumonsi dai fenomeni vitali e dai fenomeni flogistici. L' una e l' altra serie di fenomeni è subordinata alla struttura delle parti dei tessuti organizzatori nerveo, vascolare e membranoso (33 e seguenti); quindi nel modo istesso che la vitalità esprime in questi tessuti la forza della sua reazione colle forme vitali della sensibilità, contrattilità e tonicità, la flogosi accenna la sua presenza colla diversità rispettiva delle sue proprie operazioni, e costituisce per conseguenza tante forme di processo quanti sono i primordiali tessuti che costituiscono il corpo umano.

Vi sono due forme generali del processo flogistico; risipola e flemmone. Tutti i mali finora conosciuti sotto le categorie delle inflammazioni appartengono a queste due forme, sia che occupino la superficie del corpo, sia che più addentro vi affettino i visceri.

The presence or the effect of inflammation is deduced from the presence of certain characters, which Dr. Filippi says may be collected from the state of the pulse, the existence of pain, the appearance of the blood; the ability of tolerating the action of remedies, from autopsical inspection.

The treatment of inflammation calls for two orders of remedial agents. 1 External, which the author subdivides into, *a*, cold and astringent, or warm and emollient, fomentations and lotions; *b*, stimulating topical applications; *c*, vascular depletion; *d*, dry cupping; *e*, blisters of moxa and issues: 2, Internal, subdivided into 1. Therapeutical means acting on the *animal* sensibility: *a*, oleaginous—mucilaginous; *b*, stimulant; *c*, narcotic sedatives. 2. Therapeutical means, acting principally on the *organic* sensibility: *a*, diluted acids and sub-acids; *b*, Emetics and purgatives; *c*, diuretics and sudorifics; *e*, tonics. 3. Therapeutical means acting on vitality in general *(unità vitale)*; *a*, depriments; *b*, excitants.

There is too much affectation in the style, and some consequent obscurity in the language of this work; but we are, on the whole, pleased with it. We give credit to the author for the feelings that have induced him to write it; and doubt not of its having the effect of rendering the practitioner, in the part of the world he lives in, more cautious.

---

